# The Goodyear Dental Vulcanite Company

**Published:** 1881-01

**Authors:** 


### EDITORIAL, ETC.

---

*The Goodyear Dental Vulcanite Company.*—Watchful eyes, it is said, are on this long time monopoly, and it will be seen to that their application for an extension of the Cummings' patent is hotly contested. It behooves every dentist to see to it that the member of congress for his district is thoroughly posted as to the merits of this case, the injustice of the cause of the company and the importance of the question to the dental profession. We may rest assured that the company will well represent their side of the case, and it should be well taken care of by the dentists.

Since the above was written a mass meeting of the dentists of Baltimore has been held, a report of which we append as published in one of the daily papers of that city. It behooves all who feel interested in the subject, to act promptly and vigorously, for it is evident that the vulcanite company intend to use all means to gain the extension of the patent held by them. Let each dentist who has a member of congress for a patient, use what influence he has to post him on this subject.

